# Becoming futile: the emotional pain of treating COVID-19 patients

**DOI:** 10.3389/fsoc.2023.1231638

**Published:** 2023-11-08

**Authors:** Jason Rodriquez

**Affiliations:** Department of Sociology, University of Massachusetts Boston, Boston, MA, United States

**Keywords:** COVID-19, emotions, futility, health care, health care professionals, intensive care, moral distress

## Abstract

**Introduction:**

The COVID-19 pandemic has had a profoundly detrimental impact on the emotional wellbeing of health care workers. Numerous studies have shown that their rates of the various forms of work-related distress, which were already high before the pandemic, have worsened as the demands on health care workers intensified. Yet much less is known about the specific social processes that have generated these outcomes. This study adds to our collective knowledge by focusing on how one specific social process, the act of treating critically ill COVID-19 patients, contributed to emotional pain among health care workers.

**Methods:**

This article draws from 40 interviews conducted with intensive care unit (ICU) staff in units that were overwhelmed with COVID-19 patients. The study participants were recruited from two suburban community hospitals in Massachusetts and the interviews were conducted between January and May 2021.

**Results:**

The results show that the uncertainty over how to treat critically ill COVID-19 patients, given the absence of standard protocols combined with ineffective treatments that led to an unprecedented number of deaths caused significant emotional pain, characterized by a visceral, embodied experience that signaled moral distress, emotional exhaustion, depersonalization, and burnout. Furthermore, ICU workers’ occupational identities were undermined as they confronted the limits of their own abilities and the limits of medicine more generally.

**Discussion:**

The inability to save incurable COVID-19 patients while giving maximal care to such individuals caused health care workers in the ICU an immense amount of emotional pain, contributing to our understanding of the social processes that generated the well-documented increase in moral distress and related measures of work-related psychological distress. While recent studies of emotional socialization among health care workers have portrayed clinical empathy as a performed interactional strategy, the results here show empathy to be more than dramaturgical and, in this context, entailed considerable risk to workers’ emotional wellbeing.

## Introduction

As hospitals across the globe were overwhelmed by the sheer number and acuity of patients suffering from a new, deadly, and contagious virus about which little was known, the health care workers charged with treating these patients confronted a profound set of challenges that has had a lasting, detrimental impact on their social-psychological wellbeing. In the months and years since the onset of the COVID-19 pandemic, research has consistently shown that rates of work-related distress, captured by a constellation of related constructs such as moral distress, secondary trauma, emotional exhaustion, depersonalization, and burnout, which were already high before the pandemic, have worsened as unanticipated risks, higher workloads, and patient care requirements heightened the demands on health care workers (Azoulay et al., 2020; Sexton et al., 2022; Rodriquez, 2023). While numerous studies have calculated the adverse impacts of the pandemic on health care workers, fewer studies have examined the social processes that have generated these outcomes. The present study focuses on one such social process, namely, treating critically ill COVID-19 patients within the context of empirical uncertainty about how to do such a thing.

Given the absence of effective treatment guidelines or protocols at the outset of the pandemic, how did health care workers determine how to treat patients? How did those determinations contribute to the well-documented increase in social-psychological distress among health care workers, specifically the moral distress caused by giving potentially futile care? Moreover, how did treating incurable COVID-19 patients impact healthcare professionals? This article focuses on intensive care unit (ICU) staff because it is in ICUs that the most critically ill COVID-19 patients were treated, because ICU care often follows validated protocols, and because ICU workers stake occupational identities on the ability, indeed the eagerness, to give maximal care to save the most critically ill patients.

While COVID-19 intensified the dilemmas embedded in intensive care that generate moral distress among its workforce (Romero-García et al., 2022), this study shifts the focus toward how staff made sense of those dilemmas caused by the uncertainty over what they should be doing to save patients’ lives. In the absence of effective treatments, ICU staff felt moral distress, characterized by frustration and helplessness as they gave every treatment they could think of while bearing witness to an extraordinary scale of deaths they were seemingly powerless to stop. Staff described caring for critically ill COVID-19 patients in terms of disaster and wartime medicine, and experienced a range of distress markers such anxiety, depression, and post-traumatic stress. Workers’ experiences of moral distress contributed to secondary trauma, emotional exhaustion, depersonalization, and burnout. More generally, findings show that treating incurable COVID-19 patients for months on end undermined the occupational identities of ICU workers, challenging their sense of being effective health care professionals.

### Caring until it hurts

Emotional socialization among health care workers in the United States has undergone a transformation from “detached concern” to “clinical empathy” (Underman and Hirshfield, 2016; Vinson and Underman, 2020). Renée Fox’s classic study (Fox, 1959), for example, showed that medical students were trained to manage their own emotions in the clinical encounter by evincing “detached concern,” meaning to be concerned enough to be compassionate toward patients but not so concerned that clinical objectivity was impeded (Fox, 1988). Detached concern was conceptualized by Fox not as a dichotomy, but rather a duality balanced in the interactional strategies of clinicians toward patients (Cadge and Hammonds, 2012). Smith and Kleinman (1989) characterized this duality as a stance of “affective neutrality” that maintains authority over the clinical encounter. As medicine has turned toward patient-centered care, training health care workers’ emotions has shifted away from a model of detached concern and toward one of clinical empathy (Vinson and Underman, 2020).

Much of the sociological research on emotions among health care workers has emphasized its dramaturgical aspects, consistent with Hochschild’s concept of “emotional labor” (Hochschild, 1979, 1983) and Goffman’s “impression management” (Goffman, 1959) which is illuminating in some respects but limiting in others. Recent scholarship, for example, has taken an embodied practice approach, focusing less on the strategic use of emotions to manage impressions and more on the visceral experience of emotions and its connection to the practicalities of daily life given the challenges of the labor process (Cottingham, 2022). This perspective provides a more holistic understanding of emotions in health care work, as emotions are conceptualized as resources that connect across time and space, directing attention and shaping how workers’ emotional capital gets drained or reinforced given the environment. In this light, the perils of clinical empathy as something more than dramaturgy are illuminated.

Caring for, and caring about, patients in the life-and-death context of medicine combined with an organizational structure that tends to prioritize profit and operational efficiency over workers’ wellbeing leaves health care workers susceptible to work-related distress (Young et al., 2011). ICU workers are particularly vulnerable due to its demanding environment and likelihood of facing ethical dilemmas related to end-of-life care that are felt at the level of emotions (Van Mol et al., 2015). These emotions extend beyond the workplace, as ICU staff utilize emotional strategies both on-and-off the job to manage the intensity and uncertainty of caring for critically ill patients (Hammonds and Cadge, 2013).

The pandemic caused a significant amount of moral distress among intensive care unit staff (Romero-García et al., 2022). Moral distress refers to feelings of helplessness, frustration, and anger when health care workers are unsure of, unable to, or prevented from doing what they perceive to be an ethically correct action while treating a patient (Jameton, 1984, 2017). It is distinguished by emotional pain caused by constraints on the ability of a health care worker to act in a manner consistent with what they believe to be the best for patient care. While moral distress was initially intended to describe the experience of nurses bumping up against institutional constraints such as understaffing and professional hierarchies (Jameton, 1984), subsequent research has shown it affects workers across the health care sector in a wide variety of situations (Pauly et al., 2012; Rodney, 2017). Moreover, the social organization of work in ICUs generates moral distress given its higher patient mortality rate, demanding work routines, and wide range of ethical dilemmas related to end-of-life care that affect workers across occupational categories, even in the absence of a pandemic (Kon et al., 2016; Moss et al., 2016). While already relatively high among ICU workers compared to other occupations within the health care sector, moral distress was exacerbated by the pandemic and led to unprecedented levels of burnout and mental health challenges such as anxiety, depression, and post-traumatic stress (Donkers et al., 2021; Silverman et al., 2021; Guttormson et al., 2022; Romero-García et al., 2022).

The potential for moral distress among health care workers, while high even in normal times, is especially acute during a public health crisis such as COVID-19 (Hugelius et al., 2017, 2021; Cadge et al., 2021). For example, A meta-analysis of studies about front-line health care workers during pandemics including COVID-19, Ebola, SARS and H1N1 found, “consistent evidence for the pervasive and profound impact of large-scale outbreaks on the mental health of frontline healthcare workers” (Busch et al., 2021, p. 178). Because of COVID-19, a recent study showed approximately half of health care workers reported secondary traumatic stress, emotional exhaustion, or depersonalization during the pandemic, including over 2/3rds of health care workers who were exposed to patients’ death (Orrù et al., 2021). Indeed, studies have consistently shown very high rates of secondary traumatic stress among health care workers due to COVID, particularly among nurses, women, ICU workers, and others who work directly with patients (Benfante et al., 2020; Vagni et al., 2020). Intensive care workers during COVID-19 have reported very high rates of anxiety, depression, or burnout due to the overwhelming impact of the pandemic on the conditions of work (Azoulay et al., 2020; Rodriquez, 2023). The present study contributes to existing knowledge by showing how treating incurable patients contributed to moral distress among ICU workers.

### Futility

Medical futility is an inherently ambiguous, multidimensional, and context-dependent concept that nevertheless generally describes a situation when a patients’ physician has determined that a treatment offers no reasonable likelihood of benefit to the patients’ quality of life or chance of recovery, and therefore providing such treatment would be not justified given the potential risk of harm (Palda et al., 2005; Asayesh et al., 2018; Rakhshan et al., 2022). Although futility is a concept as old as Hippocrates, the ethical dilemmas in making futility determinations endure and may even be amplified as new treatments emerge that extend a patients’ life long after they otherwise would have died (Whitmer et al., 2009). The perception of futile care is closely related to moral distress among intensive care workers (Ferrell, 2006; Mobley et al., 2007; Borhani et al., 2015). A recent study, for example, of intensive care nurses during COVID-19 showed they experienced the highest intensity of moral distress when they perceived futile care (Andersson et al., 2022).

There are different ways to measure futility. Quantitative futility refers to the statistically very high likelihood that a treatment will be useless and therefore not justified (Schneiderman et al., 1990). Qualitative futility refers not to the probability of a treatment working, but to the value of the outcome even if the treatment did work. In this sense, if a treatment does nothing more than maintain total dependence on critical care to sustain life, it is futile (Schneiderman et al., 1990). Yet these conceptual distinctions belie the challenge of application to real world situations, as end-of-life care varies on a case-by-case basis and value judgments and subjective interpretations are made throughout treatment (Wilkinson and Savulescu, 2011). For instance, patients’ families may see value in continuing the life of their loved one longer than clinicians see value in continuing to treat their patient (Gampel, 2006). Furthermore, medical interventions regarded as futile by some may be regarded as important rituals with social value, especially in marking the transition between life and death (Mohammed and Peter, 2009).

A futility determination can be thought of as a professional justification to withdraw or not provide life-sustaining treatment (Wilkinson and Savulescu, 2011). Such authority is grounded in the idea of professional autonomy, meaning that health care professionals should not be forced to provide care they consider to be ineffective and potentially harmful because a patient or surrogate requested it (Gampel, 2006). As one of the most significant sources of moral distress among intensive care workers, the autonomy to resist futile care would seem to provide a buffering effect against the emotional pain inherent in critical care medicine (Asayesh et al., 2018). Disagreements between health care professionals and patients’ families about futility determinations are relatively rare in part because on most occasions the outcome is clear, but also because health care workers seek to avoid moral distress and are adept at bringing patients’ families into frame alignment with their own understanding of the situation, even as the process is recognized as shared decision-making (Rodriquez, 2021).

The variety of perspectives scholars have brought to bear on futility do not fully capture the character of futility that intensive care workers confronted during COVID-19. Rather, this article shows a process of confronting the reality that the tried-and-true treatments and procedures that save lives in the ICU were proving ineffective in this case. Futility was not so much about ethical dilemmas so much as it was about the limits of medicine itself and the limits of these actors’ medical knowledge and practices to stop their patients from dying. The emotional pain of seeing these treatments become futile was particularly severe because ICU workers often hold occupational identities, and professional authority, grounded in their ability to leverage technical-medical knowledge and practices into healing the most gravely ill patients (Freidson, 1970; Zussman, 1991).

## Data and methods

This article is based on 40 phone interviews with ICU staff that were conducted between January and May 2021. The study participants were recruited from two suburban Massachusetts community hospitals that were at times during 2020 and 2021 completely full of COVID-19 positive patients. Both ICUs were primarily pulmonary units that up until COVID-19 often treated patients who needed mechanical ventilation as a result of influenza, COPD exacerbation, congestive heart failure, or alcohol/drug withdrawal among other conditions. The sample included 15 nurses, six physicians, six nurse practitioners, six physician assistants, six respiratory therapists, and one unit coordinator. Eight of the interviewees formally supervised other staff. The purposive sample was chosen for its proportional representation of the key occupational groups and demographics who engage in direct patient care. The sample includes 29 women and 11 men, a ratio consistent with the gender distribution of the health care industry, in which 76% of job holders are women. Most interviewees were non-Hispanic white people, their ages ranged from the 20s to the 70s, and their years of experience in the healthcare industry spanned a similar range, from just starting their career to others near retirement after decades at the bedside. Nurse practitioners and physician assistants had the same functional role, while formally under the supervision of a physician they were often the highest-level worker present on the unit and had wide latitude to manage patient care independently. A referral sampling method was used to recruit participants, in which at the end of the interviews participants were asked whether they knew of anyone who may be interested in the study. Recruiting participants was not particularly challenging and many seemed eager to discuss their experiences in a formal phone interview.

The interviews followed a responsive interview format in which there was a consistent set of themes around which the interview was structured, but the specific questions differed to some degree based on the particularities of the interview (Holstein and Gubrium, 1995; Weiss, 1995; Rubin and Rubin, 2011). Each interview focused on four themes: (1) the medical treatment of COVID-19 patients including treatment decision-making and end-of-life determinations, (2) interactions with patients’ families, (3) the social experience of treating COVID-19 patients, and (4) workplace COVID-19 policies and practices. Some of the key interview questions that generated the data reported in this article include, “What has it been like to treat patients with COVID-19?” “How have treatment recommendations changed?” “How does caring for COVID-19 patients compare with treating other patients, or patients before the pandemic?” and “What has it been like to have so many patients die of COVID-19?”

The average duration of the interviews was about an hour, and they ranged between 40 and 90 min. The interviews were audio-recorded and transcribed. The data was coded with Atlas.ti. Coding was thematic and progressed from “open” to “focused” (Lareau, 2021), based initially on the four themes covered in every interview and then adding additional codes to capture emerging themes in the data. When coding was concluded, there were eight coding categories with a total of 55 subcodes within those categories. For this article, data was drawn primarily from the “Treating Patients” and “Psychosocial” code categories. Key subcodes in the “Treating Patients” code category included “Futility,” “Treatments,” and “End-of-Life.” From the “Psychosocial” code category, data came primarily from codes labeled “PTSD,” “Traumatizing,” “Exhausting,” and “Like War or Disaster.” Multiple rounds of coding took place, in which new codes were incorporated, other codes were merged, and still others were judged to be not thematically strong and were removed from the codebook. As an iterative, analytic process, codes were modified as interpretive judgments were refined (Emerson et al., 2011; Charmaz, 2014).

## Results

The first section of the results below explores what it was like for ICU staff to be treating patients without standard treatment protocols in place and how they grappled with the question of whether and when to intubate patients, given the unlikelihood of survival. The next section looks at how staff conceptualized the care they provided as futile. The third section examines the unprecedented scale of deaths and shows how staff used metaphors of war, disaster, and linked to mental health problems they would suffer as a result. The last section turns to the consequences of moral distress including emotional exhaustion, depersonalization, and burnout.

### Not knowing how to treat COVID-19 patients

Treatment in the ICU often follows a standardized path organized around protocols, checklists, and generally accepted treatment regimens that may be modified as the patients’ condition requires (Timmermans and Berg, 1997; Gawande, 2011). Yet the specialized knowledge and treatment protocols proved powerless in the early waves of COVID-19. A nurse practitioner, for example, stated that with regard to treating patients during the initial waves, “We were just kind of making it up as we go along.” Another nurse practitioner, one who was a manager of the unit, similarly reflected, “At the onset, we did not really have a treatment plan…we did not know what was going to happen day-to-day-to-day, so each day was a new day of like, ‘Well, hopefully this person is going to survive.’” Still another nurse practitioner explained that their ICU is very familiar with pulmonary patients, but COVID-19 “acts like nothing else we have ever seen,” and that “Nothing made sense. We were throwing treatments at them like you throw things at a wall to see what stuck and what would work.” A nurse supervisor noted the frustration at the absence of a viable treatment pathway: “It was in China, it was in the UK, we were like, ‘Why do we not have more of an understanding at this point how to treat the patients?’ It was frustrating.”

The novelty of the virus and the absence of effective treatments, along with its initial spread in Asia and Europe, led physicians to rely on social media to develop novel treatment regiments. One of them explained, “We got thrown into this situation where, we are treating patients based on Facebook posts, and it was true, we were. It was something that was so unique, to be so uneducated on this major crisis.” Another physician reflected, “There’s a Facebook page that people would post recommendations, studies that were being done, those sorts of things in real-time, and everybody was stuck in the same position. Nobody really knew what did or did not work.” A third physician said, “The big monster here is the social media,” and made a tongue-in-cheek reference to “WhatsApp University,” in which informal social networks were “completely inundating us with information and new knowledge.” Still another physician reflected about treating COVID-19 patients, “It was frustrating. I have to tell you, because at some point knew that nobody knew what to do” and said “It was like treating the unknown. But at some point, you know the outcome.” Social media, regarded here as both a resource and a monster, reflects the uncertainty health care workers confronted in the early stage of the pandemic and the search for viable treatments amidst a seemingly endless stream of critically ill patients.

A respiratory therapist noted how strange it was to be treating patients with unproven treatment protocols gleaned from anecdotal evidence filtered through the prism of social media platforms, rather than treating patients how they normally would with “all the things that you know you do with lung patients.” He said, “In the beginning, I’m like, ‘This is dumb. Where are we getting this advice from?’ They’re like, ‘Oh, well, we got it from Italy and what worked for them and China.’ I’m like, ‘All their patients are dead.’” A doctor noted that at first, they did not treat with steroids, “We were hesitant to use them because of kind of vague anecdotal reports from Italy that maybe they did worse with the steroids,” and it was months later that using steroids became standard practice for treating COVID-19 patients.

Clinicians throughout the units ticked through a list of all the various treatments they tried, often with resignation about their ineffectiveness. One doctor noted, for example, “We have the steroids, and we have the remdesivir, *et cetera*, I’m really not convinced any of those are game changers, at least from what I’ve seen on a personal level.” A nurse noted the options at their disposal seemed not to make a difference, saying, “They’re on a ventilator, they are not on a ventilator. Let us try this drug, let us try that drug. You’re turning them on their stomach for 16 h, on their back for 8 h, and you are trying all these things and you do feel defeated.” A physician said at one point they tried hydroxychloroquine but after a month it became clear it did not work. He said eventually they were treating patients with anything they thought may be beneficial: “Vitamin C, vitamin D, zinc, it was more like a cocktail of things that you threw at them, but it was quite frustrating because it was like recommendations changed every few weeks.”

A physician assistant supervisor explained the frustration of treating patients without effective guidelines, “The steroids, the monoclonal antibodies, the antivirals, the plasma. It’s very frustrating because, there’s a study that supports everything and there’s a study that disputes everything.” A nurse practitioner noted the frustration of “giving people advice based on advice that’s been given to you and it might be wrong.” She reflected that with COVID-19, “The standard of care has not been decided. In the beginning, I remember them saying do not take a lot of vitamin D. Do not take more than a thousand of vitamin D. And now they want you to take two-thousand of vitamin D. You know what I mean?” A nurse practitioner also went through the list of changing treatment recommendations, “First, it was the plasma and then it was the steroids and then it was the remdesivir, the chloroquine. We tried that and I’m like, ‘No. Come on. The theory itself does not even work,’ so we quit that.”

Not knowing how to effectively treat patients in the ICU with COVID-19 led to additional difficulties, especially with the critical decision about whether and when to intubate. One nurse practitioner noted her frustration because, “It’s like, no matter what we do, they die. You have somebody on a ventilator 18 days and they die, so it’s like, should you have just told the family after a week that we should stop?” There were no easy answers to this thorny dilemma. A nurse said, for example, “They’re deteriorating right in front of your eyes. And the hardest discussion was [whether] to put them on the ventilator or not because we knew right off the bat, if you put them on the ventilators, their chances of survival are next to zero.” In sum, ICU staff treated patients with everything they knew to do, and even things they were not sure of but read on social media that others had used effectively, and were left with the feeling that there was simply nothing they could do to save their patients’ lives.

### Becoming futile

Death is a part of patient care in the ICU, yet staff described something far more intense than they had ever seen. During the initial waves in spring and summer of 2020, they said, nearly all the COVID-19 patients who were admitted to the ICU died. As a doctor who supervised the ICU explained that with COVID-19, “It’s definitely been more people dying and more people dying in a way where we can see it coming as providers, sometimes a week or two ahead of time. We know what’s going to happen.” A nurse practitioner noted that “I think the hardest thing for all of us is that…in the beginning, no matter what we did, people were dying. I mean, everybody was dying.” A physician assistant reflected that, “We had so few that survived that you just saw how it was progressing. I mean, everybody would just look at each other and go, ‘Well, they are not going to make it. You saw it coming.” A nurse stated that “One of the worst things” was that “we were killing ourselves to take care of them, knowing the outcome wasn’t going to be good.”

Although most of the staff did not use the word “futile” to describe what it was like caring for these patients, what they said nonetheless described futile care. For example, a respiratory therapist explained, “There was absolutely nothing we could do. We were trying as hard and pulling every trick that we knew. There was no technique that anyone could imagine that was working. It just felt pointless and worthless.” A nurse supervisor added, “It was just physically draining because you felt like you were doing so much trying to save these people and then no matter what you did, nothing worked.” After a patient dies, a nurse practitioner told me, it was normal to ask herself if she had missed anything that could have prevented such an outcome. But in the case of COVID-19, “It’s not something that you miss, it’s that your toolbox was empty. There was nothing left to do,” she said. Another nurse practitioner said, “I mean, you try to do everything you can to save someone, and you cannot. It’s very frustrating. You feel like a failure. By the time the night’s over you are like, ‘I tried everything I know how to do and he’s dying.’” A nurse reflected, “It’s been difficult because I feel like when the patients get to the ICU, they have already reached kind of a point of no return. And I find that no matter what we do for these patients…they are not getting better.” A doctor simply said, “Sometimes you feel bad that you are doing things to patients that you know that is probably hurting them rather than being beneficial because you know the outcome.”

Some of the ICU staff directly named the care they provided to these patients as futile. A physician assistant, for example, described treatments for COVID-19 patients as “excessively futile.” When I asked what he meant by that, he explained,

It just seems like nothing works the majority of the time. These older people, we can spot them a mile away. You have the risk factors for severe disease, whether ethnicity, comorbid conditions, diabetes, your weight, renal failure. We’ll meet these patients on the floor, they’re sick enough to come to the ICU and it’s really devastating to know that they're just not going to do well with this.

A nurse practitioner similarly reflected, “As soon as they went on the ventilator, you hit a stopwatch for 10–14 days and they’d be dead just because they’d have this massive inflammatory reaction and they would die. So, it was essentially futile at the beginning.” A physician assistant, when asked to describe caring for COVID-19 patients, explained, “In one word, I would say futile” and elaborated, “They’re sitting there like a little time bomb waiting emergently to need to be intubated because we wait and wait and wait until the last minute. Then when they are just about to die, we intubate them so that they do not die, except for that they do die, but they do not for a while.” a nurse practitioner said, “With all the stress and the constant dying that’s been around, I think we are a little quicker to identify people with COVID as being futile for compressions or futile for dialysis…when people get this bad, we have missed the boat, or the boat was never there.”

Several doctors had a slightly different perspective, explaining that even if they had given treatments that could be regarded as futile there was a balance to be struck with honoring the wishes of patients and families who wanted continued aggressive treatment. When I asked a doctor about futile care, he explained that the term is out of fashion (Bosslet et al., 2015), but that “I did provide a lot, probably ineffective and possibly harmful care in prolonging life, and that does not feel good,” adding that he was “honoring at least what the family would want for them, but it’s tough.” A doctor who supervised the unit explained, “There’s a fair amount of cases that are approaching futile care, but it’s a hard balance between that and patient autonomy,” noting that patients just before they are intubated often said that they wanted “everything” done to save their life. The doctor noted, “We can limp people along for a week or two after that point. I think some of it is people just do not want to believe it either, it’s so hard.”

Other doctors described a feeling of helplessness rather than futility. For example, a doctor noted that treating COVID-19 patients was not futile in the traditional sense of the term, but rather becomes futile over time. He gave the example of futile care as continuing with aggressive care when someone has stage four cancer that has metastasized to the brain. Treating COVID-19 patients, he said, “becomes futile” because the therapeutics they had were not working, “So you are just watching people die.” Another doctor would not describe treating COVID-19 patients as an exercise in futility, characterizing it instead as reactive rather than proactive care. He said, “You’re not taking control of the situation, you are just reacting to a situation…I do feel that the disease is calling the shots and you are just responding, reacting to it, and it’s going to do what it’s going to do.” Rather than futile, he described treating COVID-19 as “helpless,” and added that he has over 20 years of experience in ICU medicine and “With COVID I still cannot predict who is going to do well, who is not going to do well, and why that is.” A nurse practitioner concurred, “The feeling of helplessness kind of takes over, and that feeling of just being overwhelmed with feeling helpless.”

### Embodied emotions

ICU staff spoke of uncertainty about how to treat patients and the helplessness they felt in giving futile care, but they did more than that. They also spoke of an embodied emotional response to the scale of death they were unable to stop. One nurse supervisor recalled:

In one night, I took five bodies from the ICU in the morgue. And our morgue was so full that we had to rent an 18-wheeler refrigeration truck. I’ll never forget when I showed up to work, and I can see this 18-wheeler. And it clearly still says Stop-n-Shop, they did remove the letters. But you could see that it said Stop-n-Shop on it, and we were using that as overfill for a morgue and just filling it up.

Another nurse recognized this as a critical moment, “When all of a sudden, you are like, ‘Oh my God, we were putting bodies in 18-wheelers.’” There was a visceral haunting to these reflections. One nurse, for example, recalled, “Even when I talk about going to the morgue, now I can smell it, it’s like a scent that does not leave you. And the bodies on top of bodies was [pause]. Yeah, it was a lot. It was a lot.” Another nurse mentioned the lasting impact of the smell, “I’ve never been to the morgue more times in my entire nursing career…And that smell. I can smell it even when I bring up the word morgue, I can just smell it. Yeah, it was a bad time.” A respiratory therapist reflected on the scale of death, “You see death, clearly, when you work in healthcare, but just to see the amount of death in such a short amount of time, I think it’s taken its toll on myself and a lot of my coworkers.” The visceral, sensory experience of repeated visits to the morgue—confronting its sights and smells—left an indelible mark, evidencing the embodied emotional impact of giving futile care.

The inability to cure patients and the resultant deaths had a lasting traumatic impact on many staff members. For example, a nurse reflected, “We all say that we all feel like we had PTSD (Post-Traumatic Stress Disorder) from it. And I definitely agree. It’s almost like there’s no real words to describe the feeling of how it really was. Especially like March, April [2020] when it was the worst of the worst.” A nurse supervisor said, “It’s traumatizing… If I knew what my job was going to be like when I first started, I never would have done this.” Another nurse reflected, “I was scared and I felt like we were not really doing anything for these patients…we kind of all felt like we were going to have some PTSD from this.” A respiratory therapist said, “This thing is unlike anything we have ever deal with before. And it’s tiring. I think a lot of us have PTSD from taking care of these patients.” A nurse practitioner remarked, “I would not be surprised at some point down the road, you end up having a lot of health care workers with PTSD.” A nurse stated, “We’ve kind of said to one another, we are like, ‘Oh, we are all going to need some serious counseling after this is done.’ And I’m like, ‘Yeah, you are probably right. We probably need it now.’ This has been tough. It’s really tested us well.” Another nurse noted the anxiety of an impending shift, reflecting that, “I’m already anticipating the night before what the next day is going to bring…never in my life have I ever worried about walking into work, or the what ifs, or what am I going to run into? It’s weird.” A respiratory therapist said, “I want to tell people every day is a nightmare. I cannot believe I have to go back there in a few hours.”

Some likened what they experienced to wartime medicine. A nurse practitioner said, “It’s like going into a war zone that you know you are not going to win.” Another nurse practitioner remarked, “Just too much time under pressure. You know, even in war they try to rotate people out to the back of the lines, that kind of thing. And this has been a constant thing for a year now.” A respiratory therapist asked if I had ever seen the movie Pearl Harbor and described a scene in which, “there’s a nurse who’s just running back and forth, being like, ‘Okay. You’re going to die. You might live. So, I’ll give you some time to stabilize.’ I remember thinking it was like that. I can only dedicate the time to people who have at least some chance of survival.” Upon learning a trauma surgeon developed a protocol to adapt ventilator tubing so one machine could ventilate multiple patients at the same time, a nurse practitioner reflected, “None of us ever thought we’d have to do that. To me, I’m not in the military. I feel like that’s something that I’d have to do if I was out in the middle of a war zone.” A nurse practitioner said of trying to explain what it was like,

It's like asking someone who was in D-Day, ‘How was it to storm the beach in France?’ or to someone in Afghanistan, ‘How was it to fight the insurgency?’ If you haven’t done it, if you haven’t lived it, it’s difficult to really understand how you feel, because you can explain the situation you’re in but not the feeling of it.

### Emotional exhaustion, depersonalization, burnout

ICU staff described in visceral terms the emotional pain caused by treating incurable COVID-19 patients. Some of them said it led to anxiety, such as a respiratory therapist who revealed, “It’s going on a year now, just in dealing with really bad anxiety and the thoughts of going to work” and that, “I cannot sleep that well because in my head I’m already amping myself up to what’s going to happen when my shift starts, so it’s been hard.” A nurse disclosed that she never wants to go to work anymore, “It’s not that I do not care. I still give my everything to my patients because that’s why I went into nursing…But over this past year, I kind of feel hopeless for everybody, and tired a lot.” A nurse practitioner similarly stated that between the volume of patients and the skill required to care for them, “it’s been coupled with overtaxed emotions and just too long. It’s been too long under pressure.”

Others suggested the inability to cure COVID-19 patients led to emotional exhaustion and depersonalization. One physician assistant, for example, described it as “frustrating, but less emotional now.” She was “more emotional” at the start of the pandemic “when you just saw all these people dying…and now, it’s not a shock.” A respiratory therapist said in much the same way that “For a while I was bothered by it [all the deaths]. Then eventually, I just wasn’t anymore.” A doctor reflected that after treating so many COVID-19 patients, “You become complacent in some ways, and you become a little bit more hardened to it…I do not think we can change the course in a lot of these patients.”

A respiratory therapist told a story that vividly captured emotional exhaustion. She was performing a terminal extubation, when the breathing tube is removed after it has been determined a patient will not survive. The patients’ family members “were sobbing uncontrollably, and I had no emotions. It did not phase me one bit. I did not care,” she said. Then the very next night she was in a different patients’ room with family members while their loved one was getting chest compressions during a cardiac arrest, “They were all sobbing and holding each other, and there was nothing for me. I told a coworker, ‘I’m done with this. I cannot get invested in these people anymore.’” She further reflected, “People were getting sick and I thought I could help them, and I was invested in them getting better…[but] it got so demoralizing when these people that you invested in died, you just cannot keep doing it.” She added, “It’s just not worth it for me emotionally and physically, it just takes too much out of you.” Her emotional exhaustion and depersonalization seemed to lead toward burnout, telling me at one point, “I do not know if I can even stay in this profession, to be honest with you.”

It is worth pointing out that the work was not just emotionally exhausting, it was often physically exhausting as well. One nurse expressed, “I know this sounds crazy, but to go into a room, whether it to be turn a pump off, or to silence an alarm. Get your full gear on, and you are doing this 100 times a day in a 12-h shift…it’s exhausting. It really is.” A unit coordinator, who was not often in patient rooms but was responsible for coordinating patient care from the front desk, explained, “It was so hard. They did everything. I mean, everything and they still could not save lives. It was so exhausting. It was like 24/7, day after day, month after month for seven, eight months.” A nurse reflected, “Just watching so much death and for a 12-h shift, not sitting down once, not going to the bathroom, not eating, working your butt off to keep them alive—and for what? Just for them to die anyways.”

Some staff members expressed sentiments consistent with burnout. One respiratory therapist said, “To watch people die when you are trying to help them, you know what I mean? It’s just so frustrating. And unless you have experienced it, you just do not know how it feels to watch somebody take their last breath or their last pulse. You know what I mean? And it’s just, it takes something out of you.” Others suggested a lost sense of purpose. A physician assistant revealed, for example, “I’ve been sitting in the parking lot saying to myself, ‘Why am I here?’ and ‘What am I even doing?’” A nurse said similarly, “My favorite saying now is, ‘I got to get out of here’… I have been a nurse for 45 years. I love my job. I do not love my job anymore. Even the regular patients, I’m tired of taking care of people.” A nurse with much less experience said that she always wanted to work in the ICU and began her career so excited to take care of the sickest patients, but confided, “Now that we have gone through COVID I feel done…I do not want to be an ICU nurse anymore.” A physician assistant stated, “I got into this profession to help people, and I do not think that we are helping people. I do not want to waste my life and profession doing something that’s futile when I can make a difference somewhere else.” A nurse practitioner took a fatalist turn when she explained, “The only thing I can console myself with is death is inevitable. You have some choice in how you go out of this world and that’s why I say pick your healthcare proxy carefully because you do not want to be tortured to death.”

Some clinicians felt hopeless. A doctor lauded the work he had previously been a part of in the ICU and said he had always felt like they “make a huge difference,” but then when COVID-19 emerged, “All of a sudden, we feel very defeated. We feel like whatever we do, it does not really make a difference.” A nurse explained that when she returned from maternity leave, she was “motivated, ready to treat these patients” and then given the scale of death, “it quickly kind of took the wind out of my sails” and “became kind of hopeless.” At first, one physician assistant supervisor appreciated the “novelty” of treating COVID-19 patients as “a heyday for ICU care,” reflecting, “It was kind of like, what you like, that’s why you work in the ICU. You do not work in the ICU to take care of borderline patients, you work to care off sick patients so that you can be aggressive and do procedures, do medications.” But then it “became frustrating because nothing, some of the conventional care that we offered, a certain percentage of the time would not get anybody better. It was very frustrating.” A nurse added, “I would not go so far as to say there was like a hopelessness, but I think we all got a little bit jaded…because you are just not seeing people survive enough. I mean, there were some, but not a lot.” Another nurse suggested a feeling of hopelessness, saying that now, “As soon as someone’s intubated, even if it’s not COVID-19, I feel like they are going to die, which is not how it used to be.”

## Discussion

While studies have shown significant increases in various measures of psychological distress among health care workers because of the pandemic, fewer have examined the social processes that have generated those outcomes (Azoulay et al., 2020; Sexton et al., 2022). The present study examined in detail one key social process, treating incurable, critically ill patients, and showed how it contributed to moral distress among ICU workers. Given the absence of effective treatment guidelines at the start of the pandemic, ICU workers gave maximal care, trying everything they could think of or that they read about on social media, but were largely ineffective. The sheer scale of suffering and deaths they were unable to stop despite the constellation of medications, treatments, and procedures at their disposal caused workers moral distress, giving way to feelings of frustration, helplessness and futility.

While medical futility is inherently ambiguous and context-dependent, hinging on probabilistic determinations of whether a treatment would benefit a patients’ quality of life or offer a chance of recovery balanced against the risk of harm, it is also one of the primary drivers of moral distress among intensive care unit workers (Palda et al., 2005; Asayesh et al., 2018; Rakhshan et al., 2022). In the case of COVID-19 presented here, futility took on a somewhat different character. Rather than providing care that was known to be ineffective from the outset of the pandemic, ICU staff went through a painful process of discovery that the typically effective therapeutics and procedures in their arsenal would not work this time around. Their own efforts became futile. An earlier ethnography of intensive care units found, much like the present study, that staff described futility as “torture” for patients, but ultimately “torture” was a self-reflective concept that referred to the emotional pain of treating incurable patients (Zussman, 1991). In this sense, the treatment of incurable patients is painful “because it challenges assumptions fundamental to the occupational identity of doctors and nurses” (Zussman, 1991, p. 115). Thus, as the bodies were being piled into 18-wheelers, ICU workers were confronting not only the limits of their own abilities, and the limits of medicine itself, they were also enduring the emotional pain of giving care that undermined their occupational identities as effective health care workers.

Related, this study extends our understanding of moral distress, which is typically understood to occur when health care providers are unable to take the correct moral or ethical action to give what they believe to be good patient care (Jameton, 1984). In the case presented by COVID-19, the feelings of frustration and helplessness typically associated with moral distress came not from conflict over taking the morally correct action so much as an inability to find the medically correct action. To be sure, uncertainty about the limits of medical efficacy comes with the territory of the ICU. But in this case, these limits were brought into stark relief by the inability of intensive care workers to effectively treat patients, undermining the sense of value and meaning workers gave to their labor, skills, and collective knowledge.

Taking a step back, it is worth noting that at times, and perhaps in the case of COVID-19, marking the distinctions between closely related concepts such as moral distress, secondary trauma, emotional exhaustion, burnout, and depersonalization, may miss the big picture. There is little doubt these concepts overlap, often come from the same causes, and generate the same outcomes. Splitting hairs over their differences feels like an academic exercise without much real-world relevance. These highly trained health care workers were giving extraordinary care to incurable patients who were nevertheless dying, underscoring the misalignment between their occupational identities and the sudden, tragic limits to their medical expertise. Furthermore, when accounting for additional factors not discussed in this article, such as the increased workload of treating COVID-19 patients (Hoogendoorn et al., 2021; Kentish-Barnes et al., 2021; Rodriquez, 2023), fear, risk, and the political rancor over the very veracity of the virus itself, perhaps it is appropriate to focus less on parsing concepts and instead simply conclude that COVID-19 has caused health care workers an immense amount of emotional pain, with significant consequences for their social-psychological wellbeing and for the health care industry more generally.

On another note, as emotional training in health professions education has transformed from “detached concern” to “clinical empathy,” research has often regarded empathy as little more than an interactional strategy (Underman and Hirshfield, 2016; Vinson and Underman, 2020). The health care workers in this study may have performed empathy in the classic dramaturgical sense (Goffman, 1959), but they also truly felt empathy toward their patients dying of COVID-19 to the extent that it caused significant distress. The emotional pain workers experienced was so intense in large part because they cared about their patients. In these findings we can see the perils of clinical empathy, as health care workers felt their patients’ physical pain so much that it led to their own emotional pain. Clinical empathy may be performative, but it is more than a performance. It is a core component of health care workers’ emotional practice (Cottingham, 2022). Empathy is a practical resource health care workers use as part of their emotional capital that gets them through their shift. In the findings presented here, clinical empathy toward COVID-19 patients depleted emotional capital and undermined the value ICU workers placed in their work.

Future research may find it fruitful to engage more fully with the contours and consequences of emotional pain in intensive care work. The findings of this study show how the limits of medical knowledge generate emotional conflicts that threaten occupational identities and contribute to moral distress, burnout, and the like. It may in fact be the key to improving the experiences of intensive care workers that improve longevity in the profession. Research could further build on this study by conceptualizing clinical empathy as an emotional practice in the daily flow of work in the health care industry (Cottingham, 2022), rather than as a performance consistent with emotional management theory (Hochschild, 1979, 1983).

Methodologically, it would have been beneficial to have integrated field observations in addition to the interviews to better capture the totality of the experience of working in an ICU during the worst of the pandemic, but restrictions on visitation made that impossible (Rodriquez, 2021). Pairing interviews with observational data often helps to fill in the gap between what people say and what they do and can more holistically evidence interpretive judgments. On the other hand, interview data provides valuable insights into how people experience emotions in ways that are often not evident through observations (Lamont and Swidler, 2014). Future research should focus on how the limits of medicine, as documented here, erode occupational identities and contribute to emotional pain, in all its forms, among health care workers.

## Data availability statement

The dataset utilized in this study are not publicly available due to privacy and confidentiality concerns. Any requests for access to the dataset can be directed to jason.rodriquez@umb.edu.

## Ethics statement

The studies involving humans were approved by the University of Massachusetts Boston Institutional Review Board. The studies were conducted in accordance with the local legislation and institutional requirements. The participants provided their written informed consent to participate in this study.

## Author contributions

The author confirms being the sole contributor of this work and has approved it for publication.
